# Hemocompatibility of styrenic block copolymers for use in prosthetic heart valves

**DOI:** 10.1007/s10856-015-5628-7

**Published:** 2015-12-24

**Authors:** Jacob Brubert, Stefanie Krajewski, Hans Peter Wendel, Sukumaran Nair, Joanna Stasiak, Geoff D. Moggridge

**Affiliations:** Department of Chemical Engineering and Biotechnology, University of Cambridge, Cambridge, UK; Department of Thoracic and Cardiovascular Surgery, University Medical Center Tuebingen, Tübingen, Germany; Cardiothoracic Services, Freeman Hospital, Newcastle, UK

## Abstract

Certain styrenic thermoplastic block copolymer elastomers can be processed to exhibit anisotropic mechanical properties which may be desirable for imitating biological tissues. The ex-vivo hemocompatibility of four triblock (hard–soft–hard) copolymers with polystyrene hard blocks and polyethylene, polypropylene, polyisoprene, polybutadiene or polyisobutylene soft blocks are tested using the modified Chandler loop method using fresh human blood and direct contact cell proliferation of fibroblasts upon the materials. The hemocompatibility and durability performance of a heparin coating is also evaluated. Measures of platelet and coagulation cascade activation indicate that the test materials are superior to polyester but inferior to expanded polytetrafluoroethylene and bovine pericardium reference materials. Against inflammatory measures the test materials are superior to polyester and bovine pericardium. The addition of a heparin coating results in reduced protein adsorption and ex-vivo hemocompatibility performance superior to all reference materials, in all measures. The tested styrenic thermoplastic block copolymers demonstrate adequate performance for blood contacting applications.

## Introduction

Polymeric elastomers have widespread uses as biomaterials, but the full scope of their potential use has not been realised. Elastomeric materials are attractive as their properties are tuneable, and their mechanical properties are similar to native biological materials. A subset of polymeric elastomers possess another property, giving them a desirable analogy with biological materials: anisotropic mechanical properties, that can be induced during processing for some types of block copolymers [1]. In particular, styrenic block copolymers can exhibit a cylindrical morphology when the fraction of styrene is approximately 18–30 % and the other block is a polyolefin such as polyethylene, polyisoprene, or polyisobutylene [2]. Styrenic block copolymers are relatively easy to process via extrusion or injection moulding, and the process of shearing or stretching during moulding can be used to align the cylinders, and this alignment is preserved upon cooling [3, 4]. The alignment of the glassy polystyrene cylinders results in macroscopic anisotropic properties. Such triblock copolymers also exhibit a form of physical cross-linking between the polystyrene domains, which improves their durability.

The opportunity to fabricate heart valve prostheses from thermoplastic elastomers shows great potential [3, 39]. Bioprosthetic valves are a current gold standard prosthesis used to treat heart valve disease. Unfortunately, their durability is a significant shortcoming [5]. Alternatively, mechanical valves have lifelong durability, but are accompanied by the requirement for anticoagulation therapy. Polymeric valves have been hailed as offering a potential solution, which may be able to overcome issues of durability while not requiring anticoagulant drug regime [6].

The anisotropic mechanical properties of native heart valves are well characterised and are recognised as essential requirements for the observed durability of native heart valves [7]. Given this premise, the use of cylinder forming block copolymers, which have anisotropic mechanical properties, is a promising application. In this study, we investigate the hemocompatibility of a selection of styrenic block copolymers. We selected one of these polymers for a prosthetic heart valve application, which was coated with a commercial heparin coating. The selected material also underwent direct contact cell viability testing.

Poly(styrene-*block*-isobutylene-*block*-styrene) (SIBS) is already in use as a biomaterial for blood-contacting applications as part of the TAXUS stent. It has also been tested as a polymeric prosthetic heart valve material [8]. Other cylinder-forming block copolymers have been used to fabricate valves for which the hydrodynamics are good [9]. In this study, we publish ex vivo hemocompatibility data for a range of saturated and unsaturated, cylinder-forming block copolymer materials which may be used in a prosthetic heart valve.

## Materials and methods

### Block copolymer samples

Styrenic block copolymers are produced by anionic polymerisation. Four cylinder-forming block copolymers were examined: polystyrene-block-polyisoprene-block-polystyrene, containing 30 wt% styrene (commercial product name Kraton D1164P); polystyrene-block-polyisoprene-block-polybutadiene-block-polystyrene (commercial product name Kraton D1171 PT), with a polystyrene content of 19 % denoted as SI/BS19; polystyrene-block-polyethylene-polypropylene-block-polystyrene (commercial product name Kraton G1730) with a polystyrene content of 22 %; and polystyrene-block-polyisobutylene-block-polystyrene having 30 % wt styrene, manufactured by Innovia LLC., denoted in this paper as SIS30, SI-BS19, SEPS22 and SIBS30 respectively. All are linear block copolymers.

Expanded polytetrafluoroethylene (ePTFE) and polyester vascular grafts, manufactured by Jostent, were used as reference materials.

We also compared our selected polymer to a commercially available glutaraldehyde-fixed Bovine Pericardium patch (trade name Peri-Guard, with Apex Processing, Synovis Life Technologies, Inc. MN, USA). In ‘Apex Processing’ the patch is chemically sterilized using ethanol and propylene oxide, and treated with 1 molar sodium hydroxide for 60–75 min at 20–25 °C. This results in <5 ppm residual glutaraldehyde, and is a method used to reduce calcification and improve the hemocompatibility of bioprosthetic heart valves.

### Sample preparation

All block copolymer samples were compression moulded at 150 °C in an electrically heated hydraulic press to a thickness of 0.3 mm. Strips of 9 × 150 mm^2^ were then cut from the polymer sheet for tests in the modified Chandler loop. Samples were sterilized with ethanol before testing.

The polymer was primed for coating by forming a cationic surface on the uncharged synthetic material. This was then heparin coated by exposing to a dilute water solution containing heparin conjugate manufactured by Corline^®^ (Uppsala, Sweden). The heparin conjugate binds to any cationic surface with very strong affinity due to the multiplicity of anionic groups [10, 11].

### Coating

To increase blood compatibility of materials, which display superior mechanical properties the use of chemical modifications like heparin coating have been studied in great detail. In the Corline heparin coating, approximately 70 heparin molecules are bound to a polyamine chain, which is in turn bound to the substrate [12].

### Chandler-loop model

**Fig. 1:**
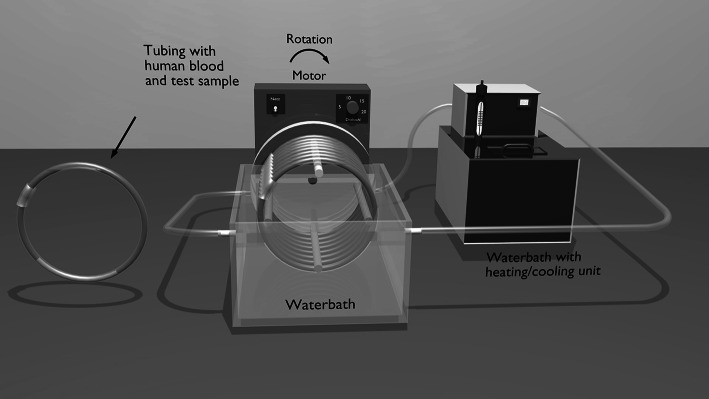
Schematic of Chandler-loop

According to ISO 10993-1 potential biomaterials for cardiovascular applications should undergo testing for their hemo- and cyto-compatibility. Experiments were performed using an ex vivo closed loop model (modified Chandler-loop) which uses a minimum of fresh human donor blood, induces physiological shear rates and has a high degree of sensitivity to material type [13–15]. The modified Chandler-loop system consists of a thermostated water bath (37 °C) and a rotating unit with attached polyvinyl chloride (PVC) loops. A strip of material is placed tightly inside a tube, the tube is filled with fresh human blood and closed to form a loop (Fig. 1) [16]. The PVC loops were coated with covalently bonded heparin (CBAS, Carmeda bioactive surface, Medtronic Anaheim, CA, USA) to minimize background activation. All tests were performed on samples of identical geometry so hemodynamic effects are eliminated.

Each loop (length: 50 cm, ID: 0.95 cm) was filled with 20 ml blood from one donor and then firmly closed into circuits with a short piece of silicone tubing outside of the loop tubing. The loops were rotated vertically at 30 rpm in the water bath (37 °C). At 30 rpm a half-filled Chandler loop generates shear rates in the blood of between 50 and 300 s^−1^ [30], which are comparable to those found in major arteries [31]. After 90 min of circulation the blood was collected in appropriate syringes [15].

### Reference materials

The hemocompatibility of the styrenic block copolymers was compared to 2 commonly used cardiovascular biomaterials: ePTFE and polyester. ePTFE is an excellent biomaterial, resulting in very little activation of the coagulation cascade [17], and is commonly used in left ventricular outflow tract reconstruction. Polyester is a thrombogenic material which is commonly used in the sewing rings of prosthetic heart valves. A single block copolymer was selected (on the basis of its desirable properties for prosthetic heart valve applications), and a heparin coating was applied to this STE. This was compared to ePTFE and glutaraldehyde-fixed bovine pericardium.

### Blood drawing

Blood was collected from non-medicated, healthy volunteers (n = 6) by venipuncture with a 1.4 mm butterfly cannula from a large antecubital vein into sterile and pre-anticoagulated containers. The blood was anticoagulated with 1.5 IU/Heparin-Natrium 25000 (Rathiopharm GmbH, Ulm, Germany). Blood sampling procedures were approved by the ethics committee of the University of Tuebingen, Gemany.

Blood from each donor was split between one donor control sample, one tubing control sample, the test item samples, and the reference samples.

### Hemocompatibility tests

Blood compatibility tests were performed using an ex vivo system according to ISO 10993-4 [15], including measures of thrombogenicity, activation of coagulation, blood cell counts, platelet activation, and inflammatory response containing complement activation and secretion of polymorphonuclear elastase (PMN-elastase) from neutrophils.

### Blood sampling

Samples were taken before addition to the Chandler-loop, and after 90 min of circulation in the Chandler-loop. 20 ml samples were divided as follows:

- 2.7 ml EDTA-blood for complement and blood cells analysis (potassium-EDTA, 1.6 mg/ml).
- 10 ml citrated blood for PMN-elastase, thrombin-antithrombin complex (TAT) and hemolysis analysis (0.14 ml citrate solution, 0.106 M C_6_H_5_Na_3_O_7_·2H_2_O).
- 4.5 ml blood in CTAD-vacutainer medium for β-thromboglobulin analysis (450 μl of 0.109 M, CTAD Becton–Dickinson GmbH, Heidelberg, Germany).

### Analyses of activation markers

The samples were centrifuged immediately at 1800 or 2000×*g* for 20 min with a cryofuge (Model 8000, Heraeus, Osterode, Germany). Plasma of the blood samples were then aliquoted in 200 μl samples and shock frozen in liquid nitrogen with subsequent storage at −80 °C for further investigations. Changes in markers of coagulation and complement activation as well as blood cell release factors were measured by commercially available ELISA kits.

Samples were analysed for β-thromboglobulin (Asserachrom β-TG, Diagnostica Stago, Asnieres, France), and thrombin-antithrombin-III complex (Enzygnost TAT micro, Siemens Healthcare, Marburg, Germany) to evaluate platelet activation and activation of the coagulation system. Adsorbed fibrinogen and adsorbed CD41 to the samples were measured using a modified ELISA method as described in [18].

Leukocyte and complement activation were detected by measurements of PMN-elastase (PMN-Elastase ELISA, Demeditec Diagnostics GmbH, Kiel, Germany) and SC5b-9 (Osteomedical GmbH, Bünde, Germany).

### Blood cell count

Cell counts were measured in EDTA-blood (potassium-EDTA, 1.6 mg/ml) immediately after sampling using a fully automated cell counter system (micros 60 ABX Hematology, Montpellier, France). Hemolysis was detected using a colorimetric assay for free plasma hemoglobin (Cyan haemoglobin test, UKT, Germany).

### Morphology

After circulation in the loop, the samples were photographed and visually inspected for thrombi. The polymer samples were incubated overnight in 2 % glutaraldehyde (Serva, Heidelberg, Germany) containing PBS (phosphate buffered saline, Invitrogen Gibco, Karlsruhe, Germany) solution and subsequently rinsed in pure PBS. The remaining water was then removed from the samples using 40–100 % of ethanol (Merck, Darmstadt, Germany) in ascending concentrations. Finally, all samples were critical point dried sputtered with gold palladium and afterwards analysed with scanning electron microscopy (SEM) (Cambridge Instruments, Cambridge UK, type 250 MK2).

### Cell viability

The direct contact viability of cells upon the material is necessary for the biocompatibility of a long term implant. We evaluated the viability of murine fibroblasts (L929) upon the materials according to ASTM standards [19]. 70 % ethanol was used as a negative control, SEPS22 and Heparin Coated SEPS22 were compared to the ePTFE and polyester graft materials, Pellethane 2363-80AE (Velox, UK), and polystyrene wells without material. Pellethane 2363 80AE underwent significant testing as a polyurethane elastomer with potential heart valve application in the 1990s, so we used this as a reference material [20, 21].

The established cell line L929 (LGC Standards, UK) was cultured directly upon the materials (n = 6), which were placed in 96 well plates for 48 h. Cell count was assessed using an MTS assay (CellTiter96, Promega, UK).

### Coating durability

The heparin conjugate covalently bonds to the polymer through a disulphide coupling unit, which is a useful marker for the XPS evaluation. Coated specimens were exposed to various environments, in vitro, for 500 h to evaluate their effect on heparin coating durability [12]. Four environments, all at 37 °C, were investigated: (1) air; (2) PBS solution; demonstrating the effect of simulated physiological conditions; (3) dynamic stretching of the material in PBS solution, at 3 Hz frequency, reaching a maximum tensile strain of 15 % using a 3-point bend geometry, with a fully relaxed minimum strain, which represented conditions of a heart valve operation, though was not intended as means of assessing the mechanical durability of the *bulk* material; (4) H_2_O_2_ [3 % (v/v)] solution demonstrating the effect of oxidising agents present in blood. The chemical constitution of the heparin coated test specimens and uncoated-control material were explored with X-ray photoelectron spectroscopy (XPS) and water contact angle measurements. XPS samples were analysed using Kratos Axis Nova XPS system with an Al Kα (1486.6 eV) source.

Three samples from each environment were analysed by contact angle measurement (seven spots) and XPS (five spots).

The concentration of heparin which leached from the coating into the PBS solution was determined using the toluidine blue method [22–26]. Polymer samples (65 cm^2^) were stored in glass vials containing 20 ml of phosphate buffered saline (PBS), pH 7.4 at room temperature. Periodically, the supernatant, (2 ml) of the PBS solution was taken and mixed with a 0.005 % toluidine blue solution (2 ml). Following heparin-dye-precipitation, the absorbance of the dye-depleted solution at 631 nm was measured and the concentration of the released heparin was calculated from the heparin calibration curve constructed using sodium heparin salt from intestinal mucosal source (Sigma, UK).

### Statistics

Results were analysed using Matlab (r2014a). A two tail ANOVA with the Bonferroni correction was used to compare normally distributed samples of equal size.

## Results

### Hemolysis and coagulation

**Fig. 2:**
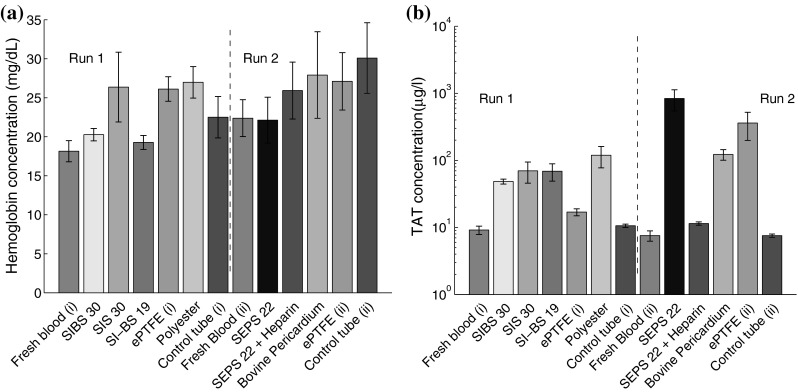
Free hemoglobin is released during hemolysis and was measured using a photometrical test. None of the materials caused significant hemolysis (**a**). Thrombin is generated in the common pathway of the coagulation cascade. We measured the concentration of thrombin-antithrombin complex. All materials activated the coagulation cascade (**b**)

In the full blood count, there were no significant differences between materials for red blood cell counts. Free hemoglobin concentration is a marker of red blood cell destruction, which would result in a material being highly incompatible with human blood. SI-BS19 and SIBS30 resulted in less hemolysis than the reference materials (Fig. 2a).

We did not observe any significant thrombus generation on the surface of the polymeric materials (Table 1). However, there was some surface thrombus generation on the bovine pericardium materials.

**Table 1:** SEM images of polymer surfaces before and after contact with blood

Sample name, (scale bar length)	No blood contact	After 90 min blood contact
SEPS22, uncoated (10 µm)		
SEPS22, Heparin coating (10 µm)		
Photograph of bovine pericardium sample after blood contact (scale marked in cm)		
Bovine pericardium after blood contact (both images). (1 mm, 100 µm)		

The coated and uncoated surfaces are macro and microscopically smooth, but have a protein coating after blood contact. The uncoated sample appears to have activated platelets adsorbed to its surface, the uncoated surface has a confluent layer of fibrin, but no activated platelets on the surface. The bovine pericardium sample after blood contact shows adherence of blood to the surface of the material. Under SEM, a mild thrombus can be seen upon the surface

Materials without surface thrombi may still cause clotting through activation of the coagulation cascade. The generation of thrombin occurs in the common pathway of the coagulation cascade. We measure the concentration of TAT in blood before and after contact with the materials. All uncoated STE resulted in more TAT generation than ePTFE and bovine pericardium, but not more than polyester. The addition of Heparin coating resulted in the least activation. Bovine pericardium also had a superior performance than ePTFE, on this measure.

The generation of thrombin-antithrombin complex occurs in the common pathway of the coagulation cascade. As such, this is a sensitive marker of activation. Bovine pericardium resulted in a lower TAT generation than ePTFE. In this case, all uncoated test materials resulted in significantly more TAT generation than PTFE. However, the addition of heparin coating to SEPS22 resulted in the lowest TAT generation.

### Platelets

**Fig. 3:**
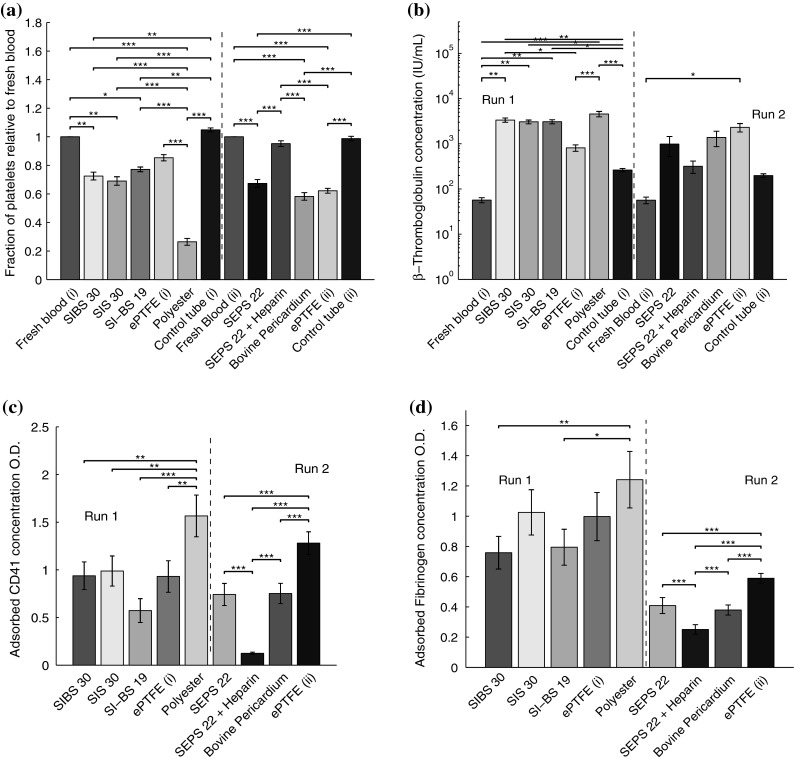
The STE and reference materials led to the loss of platelets. Platelets may be adsorbed to the material surface, aggregate to each other, or become activated, releasing α-granules. The reduction in platelet count (**a**) indicates that platelets may be adsorbed or activated. Activation of platelets leads to the release of β-thromboglobulin (β-TG) (**b**), analysed by ELISA. β-TG was significantly greater for the STE than for the hemocompatible reference, ePTFE. Adsorption of platelets to the material surface can result in the deposition of CD41 receptor protein on the material surface (**c**). The STE materials had significantly less adsorbed CD41 than the negative reference, polyester. Heparin coating of SEPS22 also reduced CD41 adsorption. Platelets and leukocytes can bind to fibrinogen which is adsorbed to the material’s surface. High levels of adsorbed fibrinogen lead to thrombus generation and localised inflammation [28]. Fibrinogen adsorption was significantly lower on the STE than on the reference materials (**d**)

When platelets come into contact with a foreign surface they can be activated. Activation proceeds through the formation of pseudopodia, adhesion, aggregation and release of platelet factors from the granules. All uncoated materials caused a drop in platelet count either through activation or adsorption of the platelets to the material. STEs, ePTFE and bovine pericardium showed similar changes in platelet counts, whereas polyester resulted in a significant reduction in platelets. If platelets are activated, then β-thromboglobulin (β-TG) is released; STEs, polyester and bovine pericardium resulted in a similar level of β-TG release (Fig. 3b). ePTFE and the addition of a Heparin coating to SEPS22 led to lower β-TG levels.

### Innate immune response

Foreign materials can active the innate immune system. Before and after circulation in the Chandler loop we measure white blood cell (WBC) count, release of PMN-elastase, and the concentration of SC5b-9.

**Fig. 4:**
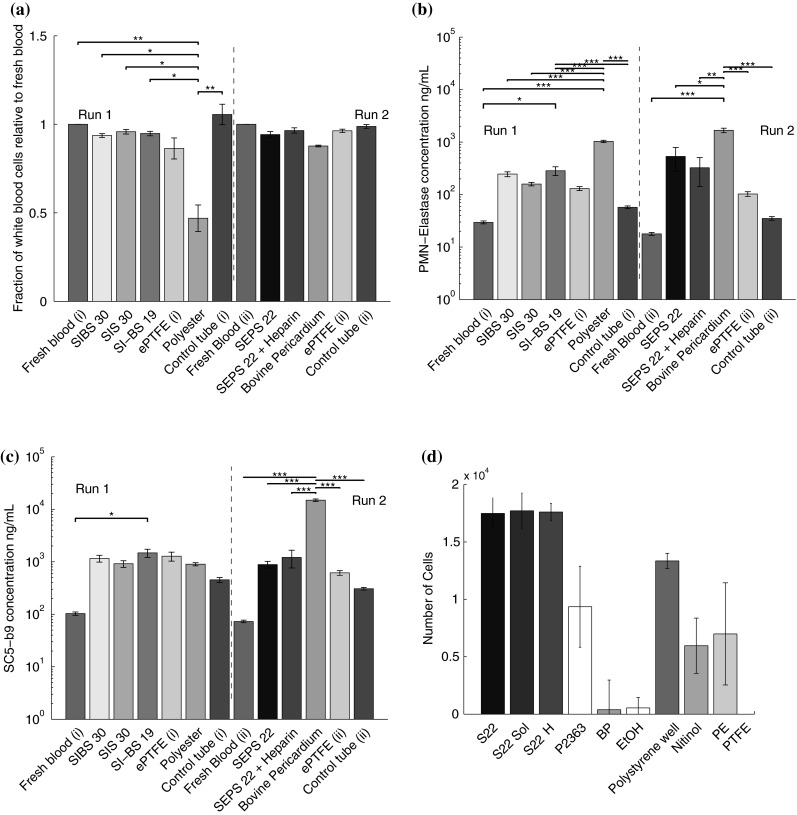
Activation of leukocytes occurs during inflammation and results in the release of PMN elastase and loss of WBC. Polyester and bovine pericardium both led to a significant reduction in leukocyte counts. None of the STEs led to a fall in leukocyte numbers (**a**). The concentration of PMN elastase was analysed by ELISA, all materials led to release, though all of the STEs were significantly less inflammatory than both polyester and bovine pericardium (**b**). SC5b-9 is associated with the final stage of the complement cascade and leads to the formation of membrane attack complexes that are used to perforate the membrane of a pathogen or cell. All materials led to the activation of the complement cascade, though all polymers were significantly less inflammatory than bovine pericardium (**c**). **d** Cells are viable on SEPS22 STE materials, with no significant differences between manufacturing methods nor coating. Direct contact cell culture of murine fibroblasts L929, cell count after 48 h using MTS assay. EtOH is 70 % ethanol, S22 Sol is SEPS22 solvent cast (10 w/v%) with toluene

The STE materials did not have a significant effect on WBC count. There was however a significant reduction (*P* < 0.05) in WBC count for samples contacted with polyester and bovine pericardium (Fig. 4a).

PMN-elastase is secreted by neutrophils during inflammation; bovine pericardium and polyester resulted in greater releases of PMN-elastase than the tested STE (Fig. 4b). The coating of SEPS22 with heparin had no effect on either of these inflammatory measures.

Plasma protein SC5-b9 is used to lyse pathogenic cells in the final stage of the complement cascade, where the alternative and classical pathways converge [27], and is a marker of the degree of inflammatory response, which may be promoted by a material. All materials led to some activation of the complement cascade, though none as much as bovine pericardium which resulted in significantly more activation (*P* < 0.001) (Fig. 4c).

### Scanning electron microscopy

Under SEM, we observed morphological differences in the proteins adsorbed on the surface of each polymer. A subconfluent protein layer was adsorbed onto the uncoated polymeric materials (Table 1). The protein layer was fibrous with some cellular entrapment and branching of fibres. In contrast, the heparin coated samples resulted in qualitatively sparser protein adsorption, in which protein fibres did not become conjoined (Table 1).

### Cell viability

We measured the viability of L929 murine fibroblast cells in direct contact with SEPS22, heparin coated SEPS22, and the reference materials. The test materials resulted in the highest growth rate of fibroblast cells upon the surface (Fig. 4d); no differences were observed between coated and uncoated SEPS22 samples.

The rapid proliferation of murine fibroblasts on the surface of the materials implies that cells are viable on the material. There was no significant difference between proliferation on compression moulded, solvent cast, or heparin coated surfaces of SEPS22, suggesting that method of manufacture is unlikely to be an issue, and heparin did not influence the fibroblasts. All methods of manufacture resulted in relatively smooth surfaces. By contrast, the reference materials were not macroscopically flat and contain ridges of approximately 1 mm, which may influence the extent to which cells could propagate. Cells were not viable on the bovine pericardium surfaces, which was likely to be due to some residual leaching of glutaraldehyde, which was used in fixation.

### Heparin coating durability

**Fig. 5:**
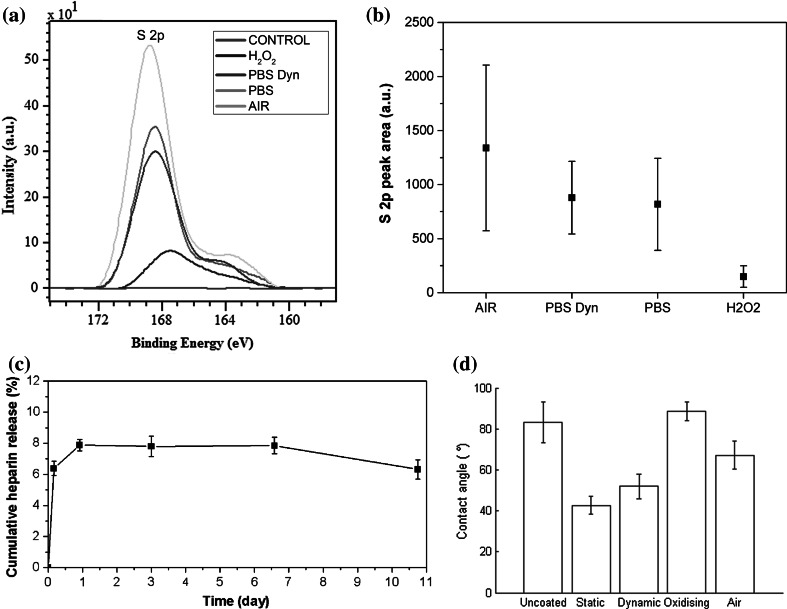
**a** Representative XPS spectra for sulphur from heparin coated polymers exposed to various environments for 500 h and for uncoated material (control). **b** Average S 2p peak area calculated from 15 measurements for each tested environment. PBS Dyn corresponds to samples stored in PBS and dynamically strained. H202 corresponds to samples stored in an oxidising environment. **c** Heparin concentration in PBS from heparin samples incubated in PBS solution. **d** Contact angle of water droplets on heparin coated samples in different environments

Heparin coated samples were aged statically in air, hydrogen peroxide and PBS, and also dynamically in PBS for 500 h. Using XPS we detected the presence of the OSO_3_ and NSO_3_ groups, which are found in active heparin (Fig. 5a). Heparin coated samples have a lower water contact angle than uncoated samples (Fig. 5d).

Each degradation method lead to a reduction in Heparin functional group concentration (Fig. 5b) and change in surface hydrophobicity (Fig. 5d). The samples which were strained dynamically have a significantly greater contact angle than those which are held statically (*P* = 0.0029) and lower sulphate group concentration, which suggests that coating has been altered or removed.

The heparin-coated materials which were dynamically strained, were not significantly different to the static samples, though there was a reduction in sulphate surface concentration for both sets of samples. There was a severe reduction in surface concentration for the samples, which were statically stored in oxidising solution. The least amount of heparin was present on samples exposed to oxidising solution. The average area of the S 2p spectra for specimens immersed in PBS solution in both static and dynamic conditions was very similar (Fig. 5b), which indicates that the dynamic stretching did not induce additional release of heparin from the surface. Furthermore, for samples stored in PBS solution phosphate spectra were also detected, suggesting that the heparin coating may have become partially covered by a layer of phosphate salts. This in turn could have contributed to a slight decrease of the sulphur peak from heparin.

Figure 5c shows that the percentage of released heparin increased sharply during the first day of the immersion before the release curve plateau at around 8 % of the adsorbed heparin. The observed heparin release was probably due to physically adsorbed heparin on the polymer surface, which diffused to the PBS solution during the first day of the experiment. As the heparin coated samples were thoroughly washed during sterilisation, and the adsorbed protein profiles are significantly different, it is unlikely that the biocompatibility improvements are a result of heparin leaching.

## Discussion

In this study we have used the Chandler loop model to characterise the ex vivo response of human blood to implantable materials.

The Chandler loop model is able to produce thrombi which are very similar to those observed in vivo [29]. Furthermore, the average shear rates generated by the Chandler loop with dimensions and rotation rate as previously described are comparable to those found in a major artery of 50–300 s^−1^ [30, 31]. For the majority of hemocompatibility measures, the modified Chandler loop was successful at removing background activation: there were only small differences between the fresh blood and control tube results for inflammatory measures and platelet activation. The ex vivo Chandler loop requires blood to be mildly heparinised prior to testing, increasing the activity of antithrombin, and so reducing coagulation. We used the concentration of thrombin as a marker for activation of the coagulation cascade, which would have been influenced by the use of heparin. Although absolute concentrations are inconsequential, the relative values are unaffected.

Day-to-day and donor-to-donor variation is a significant consideration in biocompatibility testing [32]. As such, absolute analyte levels for the test materials should be compared to the reference material results. PTFE, polyester, and bovine pericardium are used extensively in in vivo and ex vivo vascular implants and are appropriate choices of reference materials as the host response is well characterised. ePTFE is an inert biomaterial with good hemocompatibility. ePTFE is characterised by its highly hydrophobic surface and lack of functional groups, its major performance draw back as a graft material is its restenosis in small vessels. As a prosthetic heart valve material it suffers from excessive calcification and poor durability [6]. The use of polyester (Dacron) in heart valve applications is limited to the sewing ring where it is rapidly encapsulated in fibrous tissue. When used as leaflets in polymeric prosthetic heart valves the material has in vivo failure [33].

Although bovine pericardium is widely used as a graft and heart valve material with good results, there are few reports of the ex vivo hemocompatibility of bovine pericardium in the literature. In our study, the pericardium had minimal thrombogenicity and activation of the coagulation cascade, which are reflective of its in vivo performance; pericardial leaflets on prosthetic heart valves do not require major anticoagulation therapies, aspirin suffices. Among all materials, the inflammatory measurements (WBC count, PMN elastase, SC5b-9) were greatest for blood contacted with pericardium, as might be expected from a xenographic transplant. Improved pericardium processing methods, with alternatives to glutaraldehyde, could improve cell viability and reduce inflammation [34]. The pericardium used in this study did not undergo any anticalcification treatments, such as ethanol incubation, detoxification, or coating, which may be used on bioprosthetic heart valves, however the pericardium is a material in clinical use with acceptable hemocompatibility.

The STE materials did not cause significant hemolysis or activation of the coagulation cascade relative to ePTFE. Activation of platelets can be inferred from a release of β-TG, while the adsorption of CD41 to a surface is an artefact of platelet adsorption to the surface. Platelet activation was lowest for ePTFE, but was closely followed by the test STE. High levels of adsorbed CD41 may not correlate with SEM images of adsorbed platelets because they may have become washed off due to flow during the test [35]. Polyester resulted in significantly greater destruction of platelets and adsorption of CD41. The test STEs were significantly less activating and adsorbing than polyester. Contact with ePTFE resulted in significantly less β-TG release than contact with STEs. However, adsorbed CD41 levels were not significantly different between STEs and ePTFE, possibly due to the hydrophobic surface of both materials. The addition of the heparin coating mediated the activation and adsorption response, resulting in the minimum platelet activation.

There were only minor differences in hemocompatibility between the 4 types of STE. The soft block (polyethylene, polyisoprene, or polyisobutylene) is expected to coat the surface of the block copolymer materials and the minor differences in molecular architecture do not affect the surface properties (surface energy) significantly. The absence of hydroxyl and amine groups in the shortlisted biomaterials is a useful property in minimising the activation of the (alternative) complement cascade [36]. The protein adsorption profiles for the STEs are relatively similar to ePTFE and bovine pericardium so we might expect the long term biocompatibility performance of the polymers to be similar.

The adsorption of proteins is indicative of the long term performance of a biomaterial, in particular, elevated fibrinogen adsorption aids the adhesion of platelets and macrophages which can lead to a chronic, local, inflammatory reaction. SEPS22 and SI-BS19 resulted in significantly lower protein adsorption than occurred on ePTFE. Heparin coated samples resulted in a minimum of CD41 and fibrinogen adsorption suggesting that as long as the coating is present and active, superior biocompatibility may be achieved.

The polymeric materials were not cytotoxic. In the heart valve scenario, the proliferation of fibroblasts should ensure good colonisation around the sewing ring, and possibly aid the fibrous encapsulation of the leaflets which may reduce the rate of calcification. In situ endothelialisation from anastomoses over distances greater than 10 mm is unlikely. Further work would evaluate the response of monocyte-macrophage cultures to surface contact.

The addition of a heparin coating is well known to improve hemocompatibility by reducing the hydrophobicity of polymer materials [37], as well as having a biological interaction with plasma proteins [38]. The reduction in protein adsorption, as measured by assay and observed under SEM, helps to reduce coagulation and inflammatory activation, resulting in the most hemocompatible material in these tests.

These experiments are limited to 90 min of ex vivo circulation, and so the long term performance of the heparin coating was evaluated separately. The in vitro ageing indicated that some heparin may be lost during the washing phase, followed by a slow release of heparin over a period of weeks and months. Dynamically stretching the polymer or ageing in hydrogen peroxide destroyed the coating. The durability of the Corline heparin surface on pyrolytic carbon was evaluated by [12] using XPS and antithrombin binding capacity. Although they also observed a loss in heparin conjugates from the carrier chain after 3 weeks of continuous high shear flow, there was no reduction in antithrombin binding capacity.

The inactivation of heparin via ion-exchange mechanisms with endogenous cations and proteins has also been reported suggesting that long term performance is unlikely to be satisfactory even if heparin is not leached from the surface [38]. Heparin may mediate the short and medium term response and ideally lead to formation of a stable fibrous capsule, but long term performance requires further investigation, particularly for this polymeric substrate.

Patients receiving a cardiovascular implant often require a therapeutic drug regime either as a consequence of their pathology, or the implant. Patients receive at least 3 months of aspirin or another anticoagulant therapy in conjunction with a bioprosthetic heart valve implant. We considered the effect of these drugs upon the blood response through the in vitro addition of salicylic acid. In general, this resulted in a damping of the response to the tested biomaterial. However, we did observe that the combination of ex vivo aspirin and heparin coating of materials did not provide any benefit over either one used separately.

## Conclusions

A shortlist of nano-cylinder forming block copolymers were tested for hemocompatibility according to ISO 10993:4 with a view towards their application in cardiovascular devices, particularly polymer based prosthetic heart valves. None of the STE test materials elicited an adverse host response in hemocompatibility or direct fibroblast contact results. Ex vivo tests resulted in minimal hemolysis and immune system activation. The uncoated STEs which were tested performed better than polyester in terms of platelet activation and inflammation. SIBS has received considerable attention as a ‘biocompatible’ material, in this study we show that this accolade may be extended to several other STEs [39]. Furthermore, the addition of a heparin coating results in a significant improvement in hemocompatibility parameters. Although some leaching occurs in the first hours of contact, the coating is stable for at least 10 days in static solutions. The heparin coating was found to be largely still present after 500 h of testing under dynamic stretching in PBS, though was degraded after contact with oxidants.

In conclusion it is possible to rank the polymeric materials in terms of increasing activation of the coagulation cascade: heparin coated SEPS22 < bovine pericardium < ePTFE < SEPS22 = SI/BS19 = SIBS30 = SIS30 < polyester. And also in terms of increasing inflammatory reaction: heparin coated SEPS22 < SEPS22 = SI/BS19 = SIBS30 = SIS30 = ePTFE < bovine pericardium = polyester.
